# Pre-frailty factors in community-dwelling 40–75 year olds: opportunities for successful ageing

**DOI:** 10.1186/s12877-020-1490-7

**Published:** 2020-03-06

**Authors:** S. J. Gordon, N. Baker, M. Kidd, A. Maeder, K. A. Grimmer

**Affiliations:** 1 Caring Futures Institute, College of Nursing and Health Sciences, Flinders University, Adelaide, South Australia 5005 Australia; 2 Digital Health Research Centre, College of Nursing and Health Sciences, Flinders University, Adelaide, South Australia 5005 Australia; 3 Southgate Institute for Health, Society and Equity, Flinders University, Adelaide, South Australia 5005 Australia; 4 Department of Family & Community Medicine, University of Toronto, Toronto, Canada; 5 Physiotherapy Department, Faculty of Medicine and Health Sciences, Stellenbosch University, Cape Town, 7500 South Africa

**Keywords:** Frailty, Prefrailty, Middle aged, Aged, Healthy aging

## Abstract

**Background:**

There is little known about pre-frailty attributes or when changes which contribute to frailty might be detectable and amenable to change. This study explores pre-frailty and frailty in independent community-dwelling adults aged 40–75 years.

**Methods:**

Participants were recruited through local council networks, a national bank and one university in Adelaide, Australia. Fried frailty phenotype scores were calculated from measures of unintentional weight loss, exhaustion, low physical activity levels, poor hand grip strength and slow walking speed. Participants were identified as not frail (no phenotypes), pre-frail (one or two phenotypes) or frail (three or more phenotypes). Factor analysis was applied to binary forms of 25 published frailty measures Differences were tested in mean factor scores between the three Fried frailty phenotypes and ROC curves estimated predictive capacity of factors.

**Results:**

Of 656 participants (67% female; mean age 59.9 years, SD 10.6) 59.2% were classified as not frail, 39.0% pre-frail and 1.8% frail. There were no gender or age differences. Seven frailty factors were identified, incorporating all 25 frailty measures. Factors 1 and 7 significantly predicted progression from not-frail to pre-frail (Factor 1 AUC 0.64 (95%CI 0.60–0.68, combined dynamic trunk stability and lower limb functional strength, balance, foot sensation, hearing, lean muscle mass and low BMI; Factor 7 AUC 0.55 (95%CI 0.52–0.59) comprising continence and nutrition. Factors 3 and 4 significantly predicted progression from pre-frail to frail (Factor 3 AUC 0.65 (95% CI 0.59–0.70)), combining living alone, sleep quality, depression and anxiety, and lung function; Factor 4 AUC 0.60 (95%CI 0.54–0.66) comprising perceived exertion on exercise, and falls history.

**Conclusions:**

This research identified pre-frailty and frailty states in people aged in their 40s and 50s. Pre-frailty in body systems performance can be detected by a range of mutable measures, and interventions to prevent progression to frailty could be commenced from the fourth decade of life.

## Background

Successful ageing seeks to optimise health and independence [1]. Indicators for successful ageing include minimal chronic disease, physical decline or depressive symptoms, and optimised social support, social participation and economic satisfaction [2]. Bowling & Dieppe suggest that *‘a forward looking policy for older age would be a programme to promote successful ageing from middle age onwards, rather than simply aiming to support elderly people with chronic conditions*’ [3]. Successful ageing thereby avoids or delays the onset of frailty as people grow older [1].

Frailty is a broad term that incorporates a reduction in health, energy levels or cognition leading to increased susceptibility to further illness or decline in physical or cognitive function [4]. Its’ presentation is multi-factorial and varies across individuals. Frailty manifests as reduced performance and capacity in multiple body systems [5]; across physical, psychological, social [6] and cognitive [7] domains. Xue suggests that frailty is a precursor for *‘poor health outcomes including falls, incident disability, hospitalization, and mortality’* [8]. There is common agreement in the medical literature that frailty, frailty syndrome or declining function are associated with increased age and that prevention of frailty is a positive outcome of successful ageing.

A number of tools have been proposed to detect frailty in community dwelling older people [9]. These tools variably include data derived from self-reports, direct observations or measurement of performance, and clinical assessments. Examples of community-based frailty assessment tools are:

- self-report (PRISMA 7 questionnaire [10]; Groningen Frailty Index [11, 12]);
- self-reports and objective measurement (Edmonton Frail Scale [13]; Gérontôpole [14]; Frail Non-Disabled (FiND) Scale [15], Fried frailty phenotypes [16]); and
- subjective clinical determination of a person’s frailty state (Clinical Frailty Scale (CFS)) [4].

Most of the frailty assessment instruments include one or more elements of the five Fried frailty phenotypes; unintentional weight loss, feeling exhausted, weak grip strength, slow walking speed and low levels of physical activity [16]. While frailty is multi-factorial there is evidence that decline in physical function precedes cognitive decline [17–20]. A 10-year longitudinal study has provided evidence that slow gait or low handgrip are predictors of cognitive decline [21]. Hence the use of a physical based frailty tool such as the Fried phenotype was considered appropriate for pre-frail and frailty assessment of middle-aged people who are less likely to have cognitive decline. As well the Fried frailty phenotype provides an accepted definition of pre-frailty when one or two of the elements of the Fried frailty phenotype are detected [16]. It is not expected that people aged 40 to 75 years will be mostly frail rather the intent is to identify and understand factors which contribute to pre-frailty and ultimately may progress to frailty. Previous research developing frailty indicators has largely missed the opportunity to identify contributors to pre-frailty and frailty in the middle years by the consistent exclusion of younger people.

The World Health Organisation (WHO) proposed a trajectory of age-related disability in 2001, which hypothesised that without intervention, declining function could be detected in middle age, defined as prior to 60 years [22]. More recently, Theou et al. [23] found that age was not a significant predictor of frailty in a large Irish community-dwelling population aged 50+ years. Hanlon et al. [24] assessed frailty phenotype data extracted from the UK Biobank on 493,737 people aged 37–73 years, and identified one or more frailty markers across all ages, and both genders. Globally reports of pre-frailty and frailty using the Fried phenotype have reported that in England 3.9% of 8095 people aged 50 to 65 years were frail and 31.6% prefrail [25], across 10 European countries of 9074 people aged 50 to 64 years 4.1% were frail and 37.4% were prefrail [26] and in Taiwan 33.3% of 12 people aged 50–64 years were pre-frail [27]. The progression from pre-frailty to frailty in older adults has been reported recently [28, 29]. These authors suggested that self-reported and test-based measures should be combined to determine sensitively the level of frailty.

For successful ageing to become a reality in policy, public health, health promotion and clinical practice, a better understanding is required of how pre-frailty manifests and progresses to frailty, and how pre-frailty might be mitigated by population-based interventions. This paper explores the occurrence of Fried frailty phenotypes in Australians aged 40–75 years living independently in the community. It also reports factor analysis of 25 predictor variables from not frail to pre-frailty and frailty in this group.

## Methods

### Reporting standard

This paper has been written in accordance with the Strengthening the Reporting of Observational Studies in Epidemiology (STROBE) Statement: guidelines for reporting observational studies (Appendix I).

### Ethics approval and consent to participate

This study was approved by the Southern Adelaide Clinical Human Research Ethics Committee (South Australia) approval 391.16. This paper conforms to the principles embodied in the Declaration of Helsinki. Return of online surveys implied consent. All participants provided written informed consent prior to objective assessment.

### Consent for publication

Written informed consent was obtained for use of data for publication from each participant.

### Aims

This paper reports:

1. the classifications of not frail, pre-frail and frail by age and gender for community-dwellers aged 40–75 years using the Fried phenotype [16] and
2. factor analysis of 25 possible contributing health factors to determine contributors to the three classifications of frailty.

### Study design

*C*ross-sectional observational study.

### Setting

All research was conducted in Adelaide, South Australia at venues provided by Aged Care Housing Group, Flinders University, the Councils of Marion, Holdfast Bay and Salisbury, and National Australia Bank.

### Recruitment

Invitation via three metropolitan councils, one national bank and one university in Adelaide, Australia invited participation from people aged 40 to 75 years living independently at home. Participants were screened using physiological measures on the day of assessment and if considered unwell were excluded. Data were collected at each site for 4–6 weeks between January 2017 and June 2018 using a similar approach to the biennial Tokyo Metropolitan Institute of Gerontology healthy aging survey [30].

The development of the assessment protocol [31] and the method of data collection [32] for this study have been reported elsewhere. A summary is provided here for the convenience of the reader.

#### Data collection

Data was collected between January 2017 and July 2018 on an extensive range of evidence-based health and frailty measures. These measures were identified from an extensive review of measures related to functional decline [31]. Self-reported data was collected via online or hard copy survey prior to objective assessment and included:

- demographic data (age; gender; ethnicity; main language spoken at home; marital status; housing; employment; income; highest level of education; diagnosed health conditions; health concerns; alcohol and tobacco use; current medications; hospitalisations, emergency department presentations, falls and ‘near-miss’ falls in the past 6 months; unintended weight loss; appetite; participation in community activities); and
- continence [33], sleep quality [34], nutrition [35], hydration, usual activity patterns [36] and psychological distress using validated instruments [37] (see Table 1).

**Table 1:** Thresholds/ cut points in elements relevant to expected performance (Bolded measures indicate the way that the Fried frailty phenotype attributes were calculated)

Frailty measures	Calculation	Threshold for poor performance (referenced for published norms)
Walking speed	**Six Minute Walk Test** [38]	**>80th% distribution of differences between predicted and actual 6MWT** [38]
Grip strength	**Dominant hand grip strength measured in sitting** [39, 40]	**< 10th% age-gender norms** [40]
Self-reports of unintentional weight loss	**Yes/ No**	**Yes (1)**
Self-reported physical activity [36]	**Accumulate 150 to 300 min (2 ½ to 5 h) of moderate intensity physical activity, or 75 to 150 min (1 ¼ to 2 ½ hours) of vigorous intensity physical activity, or an equivalent combination of both moderate and vigorous activities, each week.**	**1 = Less than median recommended time per week spent walking, and no moderate or vigorous activity** [36]
K10_tiredness score (Q. 1) [37]	**Single response item scored 1–5, with 1 = none; 5 = all of the time**	**4 or 5** [37]
Modified Functional Movement Screen (FMS) elements (0–3, with 0 being pain precluding activity, 1 being unable to attempt test, 2 being partial attempt; 3 successfullycompleted test) [31, 41]	Sum of scores for deep squat, hurdle step, in line floor lunge, opposite side arm / leg extension in four-point kneeling	≤12
Capacity to walk a flight of stairs [38]	Self-report Yes / No	No (0) [38]
GPCog [42]	Summed scores	≤8 [42]
BMI [43]	Underweight	≤18 [43]
BMI [43]	Overweight / obese	≥26 [43]
Lean muscle mass [44]	Calculated for males as 0.407* weight (kgs) + 0.267* height (cms)- 19.2; and for females as 0.252* weight (kgs) + 0.473* height (cms)- 48.3 [44]	≤24.5 [44]
Chronic health conditions	Total number of current chronic conditions	≥1
Health concerns	Any	1
Pain	Any pain * length of time suffered (years)	≥2
Total nutrition score [35]	Sum of (Yes scores to daily consumption of 5+ serves vegetables; 2+ serves fruit; mostly eat wholegrain or alternative grains; one serve day meat or alternatives; 2 serves dairy, limited intake of sugary drinks, processed foods and takeaways)	≤6
Water intake [35]	Not answering ‘plenty’	0 [35]
Modified K10 [37]	Total score minus exhaustion component (Question 1)	≥12
Health concerns	Any	1
Continence concerns [33] (score 1 for each reported problem * degree of bother) [bother scored 1 = not at all to 5 = a lot]	Total score of urge incontinence, stress incontinence, frequency, problems emptying bladder, urinary leakage, discomfort, bulging pelvic floor, faecal incontinence	≥3
Unplanned health service use in past 12 months	Sum of number of unplanned hospitalisations, Emergency Department contacts	> 1
Living status	Alone	1
Total sleep quality score (PSQI) [34]	Summed scores	≥8 [34]
Near miss falls in last 6 months and/or falls in the last 6 months	yes, no	1 = yes (any)
Balance for 5 s (eyes open, standing on R or L leg) [45]	5 s is compliant for each leg (summed for Right + Left leg)	1 is < 10 s [45]
Balance for 5 s (eyes closed, standing on R or L leg) [45]	5 s is compliant for each leg (summed for Right + Left leg)	1 is < 10 s [45]

Objective data was captured in two-hour sessions, using multiple measurement stations. Risk screening compared physiological measures (blood pressure, blood glucose, heart rate, blood oximetry, temperature and respiratory rate) to expected values [46]. Participants with measures outside the expected values were excluded from further participation and referred to their doctor or the assessment was modified. Those without safety risks proceeded to measurement stations for anthropometry [43] (height and weight from which BMI and lean muscle mass [44] were calculated); audiometry [47]; balance [45]; cognition and memory [42]; upper limb dexterity standardised by gender and age [48]; 6 min walk test (standardised by Australian norms) [38]; exertion and dyspnoea [49]; foot sensation and skin health [50]; grip strength in sitting for dominant and non-dominant hands (standardised for age and gender) [39, 40]; lung function and lung ratio compared with predicted lung ratio (standardised by age and gender) [51]; muscle strength, core trunk stability and flexibility [31, 41]. Table 1 reports the health and frailty measures assessed with the expected normal values.

#### Outcome measure

The Fried frailty phenotype (2001) [16] was calculated from:

- Unintentional self-reported weight loss of > 10 lbs. (≥4.5 kg) or ≥ 5% of body mass in the last year;
- Weakness, assessed as sitting dominant handgrip strength, which was below 10th% normative values extracted from the age-gender-specific dataset reported in Table 2 in Dodds et al. [39]. These values were derived from over 60,000 grip strength measures reported in 12 British population studies [39];
- Exhaustion (self-report) from Question 1 of the K10 instrument ‘About how often did you feel tired out for no good reason?’ scored as 4, most of the time or 5, all of the time [37];
- Slow gait, determined by individual Six Minute Walk Test (SMWT) scores slower than standardised values for Australians [52] (calculated for males as 1005-(5.68 * age in years) + (0.89 * height in cm); and for females, 602 - (2.97 * age in years) + (2.05 height in cm) - (5.50 * BMI) by more than; and
- Low physical activity less than the median recommended amount of time spent walking per week, and no moderate or vigorous intensity physical activity each week [36].

**Table 2:** Percentage of pre-frail and frail participants with each component of Fried frailty phenotype

Attribute	Pre-frail	Frail	N
Unintentional weight loss	19 (7.4%)	3 (25.0%)	22
Poor grip strength	86 (33.6%)	11 (91.7%)	97
Low physical activity	36 (14.1%)	5 (41.7%)	41
Exhaustion	41 (16.0%)	8 (66.7%)	49
Slow walking speed	143 (55.9%)	12 (100%)	155

Individuals were identified as ‘not-frail’ if they demonstrated no frailty phenotype attribute, pre-frail if they exhibited one or two attributes, and frail if they exhibited three or more attributes [16].

#### Management of health measures

Psychological distress (anxiety and depression) was calculated from the sum of Questions 2–10 of the K10 instrument [37] (minus Q1 (exhaustion)). This modified total was split at the median value. A composite measure of trunk stability and muscle strength was calculated using a the Functional Movement Screen [41] modified by Gordon et al. [31] and included the sum of scores for the squat, hurdle step with left and right leg, floor lunge with left and right leg, and two point kneeling with opposite arm and leg extended (left leg, right arm; right leg, left arm). This score was cut at the median value. A total nutrition score was calculated as the sum of Yes scores for per-day consumption of at least one serve of meat, chicken, fish or substitute; at least five serves of vegetables; at least two serves of fruit; eating mostly wholegrain or high fibre cereals; eating weekly alternative cereals; at least two serves of dairy; and limiting sugary drinks, processed foods, and junk foods. A composite continence concern score was calculated as the sum of Yes responses for any of urge incontinence, stress incontinence, frequency, problems emptying bladder, urinary leakage, discomfort, bulging pelvic floor, faecal incontinence, with each Yes score multiplied by the amount of ‘bother’ (scored 1 = not at all to 5 = a lot) [33].

Population thresholds/ norms were applied to the measures for sleep quality, BMI (underweight/ overweight/obese), dexterity, cognition and memory, perceived exertion and dyspnoea, lean muscle mass, and lung function. The median value of the remaining continuous variables was determined as the cut point for analysis (trunk stability and muscle strength; continence concerns; pain; chronic health conditions; nutrition). Table 1 reports the cut point for each measure.

#### Sample size calculation

It was not possible to calculate a sample size, as there was no precedent for effective recruitment processes for this type of study, and no informed anticipation of volunteer rate. Thus as many people as possible were sought.

#### Statistical methods

Differences in gender proportions and mean age (Standard Deviations (SD)) were determined between the three Fried frailty phenotype classifications, using chi square (chi^2^) test of proportions, and Analysis of Variance (ANOVA), respectively. Significance was determined at *p < 0.05*.

Factor analysis was applied as a method to identify latent variables that may not be measured directly, by collapsing large numbers of variables into correlated clusters [53]. Each factor identifies a set of variables which cluster together, to describe a latent construct of frailty. We believe that the different clusters of variables represented within each latent construct (factor) reflect the multifactorial nature of frailty. Principal component analysis and varimax rotations were used to identify latent factors, and important component variables in each factor were identified as having weightings ≥0·30. The factor in which each variable had the highest weighting was generally the one in which that variable was retained. However where a variable had similar weightings across more than one factor, decisions regarding its best placement were made on an a priori clinical and theoretical basis. This was relevant to the context of the other variables loading onto the same factor and included the epidemiology of aging [54, 55] and the WHO trajectory [22].

The weightings of the variables that loaded onto each factor were summed to provide new (latent) attributes of frailty. Factors were named for the characteristics of the included variables in terms of how they described frailty. Mean scores (SD) were calculated for each factor, in each Fried frailty phenotype category [16], and Analysis of Variance Models (ANOVA) were applied to test for differences between consecutive frailty categories. SAS Version 9.4 was used for analysis [56].

Item weightings in factors were multiplied by 100 for computational ease. Per-participant scores were calculated for each factor (frailty attribute) by multiplying each participant’s at-risk score (0 or 1) by the weighting for each variable included in each factor, and then summing the weightings. For example, if a participant had zero risk for a variable, the contribution of that variable to their overall score for that frailty attribute was zero (0*loading). Conversely, if a participant was at-risk for that variable, its contribution to the total score for that frailty attribute was 1*loading [57].

For the factors that showed significant differences between consecutive frailty categories, Receiver Operator Characteristic (ROC) curves were calculated [58]. As there is no robust information on prevalence of pre-frailty or frailty in Australian community-dwellers aged 40–75 years, we assumed that the ratio of positive and negative cases was unknown. The ROC curves tested the predictive capacity of each factor to detect individual frailty states, and to differentiate between them. The findings were reported as the Youden Index (a summary measure of the predictive capacity of the ROC curve) sensitivity, specificity, criterion value (best cut point trade-off) and area under the curve (AUC (95%CI)) [57]. Predictive capacity is determined as the ability of a test to distinguish between different health states (for instance not frail and pre-frail). The higher the AUC, the better the test in discriminative capacity. For instance, when AUC is 0.75, there is 75% chance that test can distinguish between different health states, however when AUC approximates 0.5, the test has no discriminative capacity and is consequently of no value. AUC was significant if the lower 95%CI did not include 0.5 (which is an indicator of no predictive capacity) [57].

## Results

### Participants, gender, age and frailty phenotype classifications

There were 656 participants (67% female; overall mean age 59.9 years, SD 10.6). The sample compared well with local population estimates of age and gender, and all socioeconomic indices were reflected in the reported postal area codes (46% metropolitan) [51]. Using the Fried frailty phenotype categories 59.1% (*N* = 388) were classified as ‘not frail’, 39% (*N* = 256) as ‘pre-frail’ (one or two components), and 1.8% (*N* = 12) as ‘frail’ (three or more components). No participant reported all five Fried criteria, with *N* = 187 reporting one (28.5%), *N* = 69 reporting two (10.5%), *N* = 9 reporting three (1.4%) and *N* = 3 reporting four (0.5%).

Considering the individual components of the Fried frailty phenotype, the two most common components were slower than expected walking speed identified for *N* = 155 (23.6% sample), and poor grip strength identified in 14.8% sample (*N* = 97). Exhaustion was reported by *N* = 49 people (7.5% sample), unintentional weight loss by *N* = 22 (3.4% sample) and low regular exercise patterns by *N* = 41 (6.2% sample).

The frequency of reporting of each component of the Fried phenotype in the ‘pre-frail’ and ‘frail’ criteria is reported in Table 2. Whilst the percentage of people with each Fried phenotype component is smaller in the ‘pre-frail’ group compared to the ‘frail’ group, this table highlights the consistency of reporting of each Fried criteria in the ‘pre-frail’ group.

There were no gender differences (*p > 0.05*) within frailty categories with similar numbers of males and females being classified as not frail, pre-frail and frail (61.4% female, 54.6% male being not frail; 37.4% female, 42.2% male being pre-frail; 1.1% female, 3.2% male being frail *(p > 0.05*)). There were no significant age differences between the Fried frailty categories (*p > 0.05*) (‘not frail’ mean age 59.9 years (SD 10.4 years); ‘pre-frail’ mean age 60.1 years (SD 11.2 years) and ‘frail’ mean age 59.2 years (SD 8.3 years)). Considering pre-frail and frail classifications in age groups, in the 40–49 year olds, 45.0% were pre-frail, and 1.4% were frail; in the 50–59 year olds, 34.6% were pre-frail, and 1.9% were frail; in the 60–69 year olds, 34.3% were pre-frail, and 2.4% were frail; and in the 70–75 year olds, 44.4% were pre-frail, and 1.3% were frail. This indicated that participants of either gender and any age could demonstrate attributes of frailty.

### Factor loadings

Nine possible factors were identified by factor analysis, of which seven had at least two of 25 predictor variables loading strongly onto them, explaining 84.6% total variance (Factors 1–6, Factor 9 (renamed Factor 7 after discarding the original Factors 7 and 8). All seven factors were retained. Table 3 reports the factors, the component variables in each, and the weightings applied to each important variable in each factor.

**Table 3:**
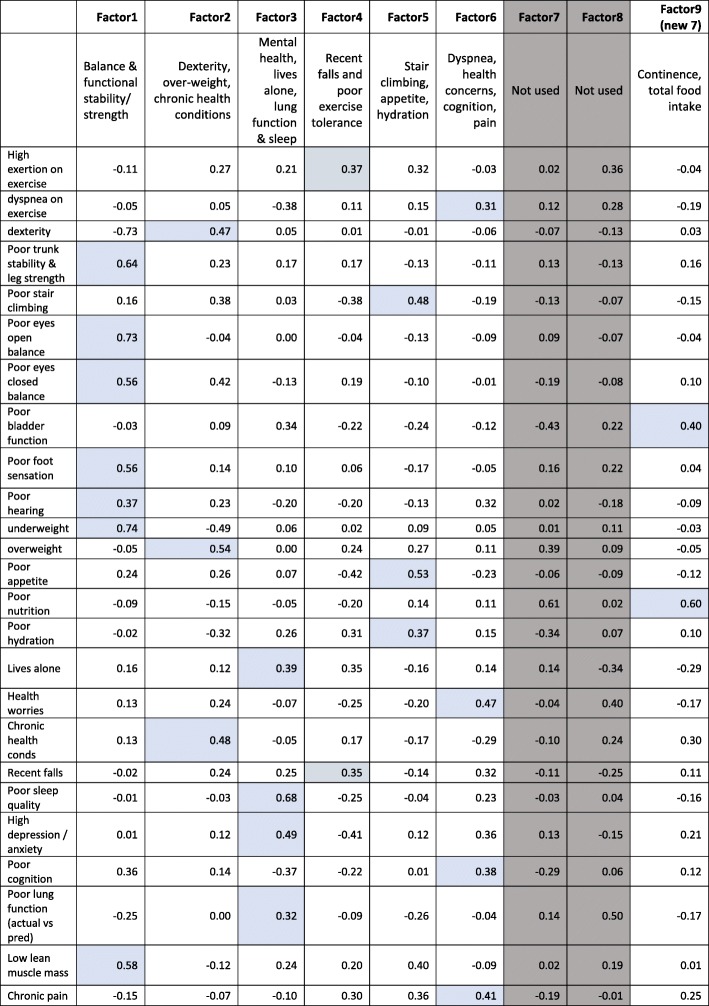
Factors, descriptions and important variable loadings

**Key:** Two factors that are greyed out are redundant. The shaded cells indicate the variables that were included in each factor, and their weightings in the latent frailty attributes (factors)

Factor 1 described safe ambulation, balance and functional stability (encompassing poor dynamic trunk stability and lower limb strength, poor balance (eyes open and shut), poor foot sensation, and being underweight (explaining 23.6% of the overall variance). Factor 2 encompassed ill-health and its sequelae (poor upper limb dexterity, being overweight, suffering chronic health conditions) (explaining 12.9% overall variance). Factor 3 dealt with managing personal circumstances (high psychological distress, having health worries, poor lung function, living alone and poor sleep quality) (explaining 11.5% variance). Factor 4 encompassed mobility constraints and safety (high perceived exertion on exercise, falls and/or near miss falls) (explaining 10.7% variance). Factor 5 described other mobility and wellness constraints, including difficulty climbing stairs, poor appetite, inadequate hydration (explaining 10.2% variance); Factor 6 described deteriorating bodily function, in terms of dyspnoea on exertion, poor cognition and memory, health worries, and chronic pain (describing 8.9% variance); and Factor 7 (initially Factor 9) described issues with continence and adequate nutrition (explaining 7.1% variance). NB The original Factors 7 and 8 had been removed from analysis (see shaded columns in Table 3).

There were significant differences in mean total factor scores across the Fried frailty phenotype categories for Factors 1, 3, 5 and 7. Table 4 reports the mean factor scores (SD), number of participants contributing to each frailty phenotype category, and ANOVA statistics (*F* value (degrees of freedom_(df)_), *p* value). Where significant differences in factor scores were identified between consecutive frailty categories, scores were bolded, and cells highlighted. In all significant factors (as hypothesised), the mean scores were higher in subsequent frailty categories, indicating that participants in the higher (more at-risk) frailty category contributed higher (at-risk) scores. There were no significant differences in factor scores between frailty categories for Factors 2, 4 or 6. Factors 1 and 7 mean scores were significantly different across all frailty categories; whilst Factors 3 and 5 mean scores were significantly different only between pre-frail and frail categories (with no differences between the not-frail and pre-frail categories).

**Table 4:**
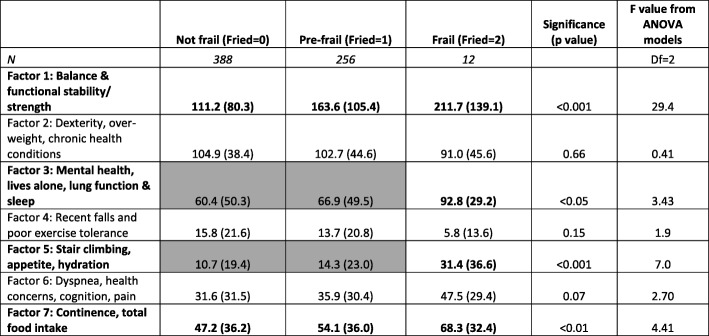
The number of participants and mean scores (SD) for the seven latent frailty attributes (factors), for the three Fried categories, and ANOVA statistics (*F* value, *p* value) for comparison across categories

**Key:** The paired frailty categories with significant differences in factor scores are bolded, and the factors for which adjacent categories were not significantly different are shaded grey (with the significantly different category bolded) (df = 2)

### Predictive capacity

Receiver Operator Characteristic (ROC) curve outputs are reported in Table 5. Based on the ANOVA findings, comparisons were made between predictive capacity for consecutive pairs of frailty categories for Factors 1 and 7 (not-frail compared with pre-frail, and pre-frail compared with frail), and between pre-frail and frail for Factors 3 and 5. Factor 1 moderately discriminated between no-frail and pre-frail states (Area Under the Curve (AUC) 64%, cut point 114), however it differentiated less convincingly between ‘pre-frail’ and ‘frail’ states (AUC 60%). Factor 7 was modestly predictive of pre-frail state from frail state (AUC 61%, cut point 40) but was poorly discriminatory of not-frail and pre-frail states (AUC 55%). Both Factors 3 and 5 differentiated moderately between pre-frail and frail states (AUC 65%, cut point 68.3; AUC 0.63%, cut point 37 respectively). In summary, only the factor measuring balance & functional stability/ strength (Factor 1) convincingly discriminated between not-frail and pre-frail states.

**Table 5:** Receiver Operator Characteristic (ROC) curve statistics

	Comparing not frail with pre-frail	Comparing pre-frail with frail
**Factor 1**		
AUC	0.64	0.60
95%CI	0.60–0.68	0.53–0.66
p	< 0.01	0.33
Youden index	0.23	0.12
threshold score	> 114	> 213
Sens	62.9	58.3
Spec	59.8	64.8
**Factor 3**		
AUC		0.65
95%CI		0.59–0.70
p		< 0.01
Youden index		0.37
threshold score		> 68.3
Sens		75.0
Sp		61.7
**Factor 5**		
AUC		0.63
95%CI		0.56–0.69
p		0.25
Youden index		016
threshold score		> 37
Sens		33.3
Spec		92.2
**Factor 7**		
AUC	0.55	0.61
95%CI	0.52–0.58	0.55–0.67
p	< 0.05	0.17
Youden index	0.07	0.16
threshold score	> 40	> 40
Sens	58.9	75.0
Spec	48.2	41.0

**Fig. 1:**
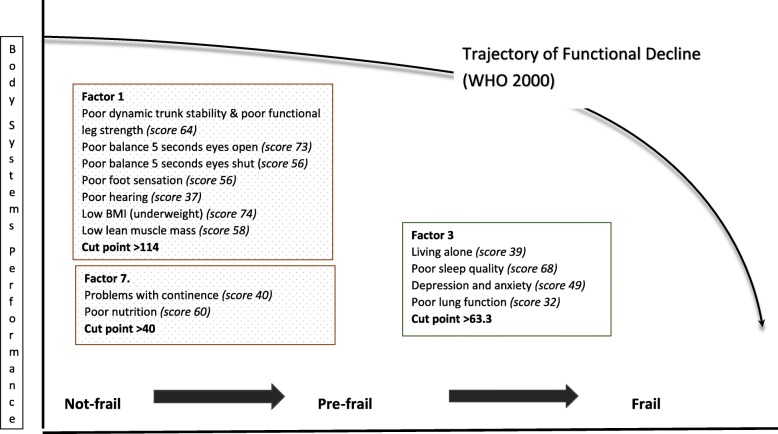
The factor descriptors able to discriminate between not-frail, pre-frail and frail status

In summary Fig. 1 presents the factor descriptors that significantly discriminate between not-frail, pre-frail and frail status.

## Discussion

To our knowledge, this is the first Australian study to report on pre-frailty in presumed healthy, independently living community-dwellers aged 40 to 75 years. We used an established frailty phenotype with two objective components (grip strength, walking speed) and three self-report measures (unintentional weight loss, physical activity, exhaustion) [16]. This phenotype was developed on people aged 65+ years and has been reported to sensitively identify pre-frailty and frailty states in this population [16]. Our research indicates that using this frailty phenotype, pre-frailty is detectable as a separate state of health to ‘not frail’, or ‘frail’, in younger community dwellers aged 40–75 years. Moreover, neither age nor gender was significantly associated with any frailty state. Thus, our findings not only add support to the theoretical WHO trajectory of frailty [22], but they also suggest that frailty is not necessarily a corollary of older age.

Our frailty rates are comparable with those published recently from analysis of data from a large UK biobank, reporting on 493 737 people aged 37–73 years (3% frail, 38% pre-frail, and 59% not frail [24] (compared with our Fig. 1.8% frail, 39% pre-frail, and 59.2% not frail). Nevertheless, we were alarmed by the prevalence of ‘pre-frailty’ in our sample and its occurrence in people aged 40–59 years. A designation of ‘pre-frailty’ requires one or two components of the Fried phenotype to be present. Given that the two most common components in the Fried frailty phenotype in our sample were related to poor grip strength, and slow walking speed, we hypothesised that at least one of these would be present in most people who were classified as ‘pre-frail’. Whilst Table 2 supports this hypothesis (33.6% pre-frail people had poor grip strength, and 55.9% had slow walking speed), this table also shows that the other three frailty components (exhaustion, poor exercise behaviours, unintentional weight loss) were found in some people designated as ‘pre-frail’.

This study essentially correlated multiple indicators of frailty, by assembling an outcome measure from the five components in the Fried frailty phenotype and testing it against latent variables constructed from a range of other measures reported in the literature as relevant to frailty. The Fried frailty phenotype components were not double counted in predictor variables. For instance, the total K10 psychological distress score was modified by removing Question 1 because this question about exhaustion was already accounted for in the Fried criteria. The seven latent frailty factors provided new information on clusters of frailty attributes, particularly as the components in each factor were justifiably related on a priori bases. For instance, the best predicting factors for pre-frailty (Factors 1 and 7), accounted for 30% variance, combining attributes of safety and stability (poor dynamic trunk stability and lower limb strength, poor balance, poor foot sensation, being underweight (Factor 1) and continence and nutrition (Factor 7)). Factor 3, the only one which significantly (albeit moderately) predicted frailty from pre-frailty in this sample, accounted for 11.5% variance, dealing with important factors associated with poor mental state i.e. living alone, high psychological distress, poor lung function and poor sleep quality.

The Fried frailty phenotype is based on two objective measures (grip strength, walking speed), and three self-report components (exhaustion, usual exercise behaviours, unintentional weight loss) [16]. It was developed for, and tested on, people aged 65 years and older, and one or more of its elements have been incorporated into other frailty descriptors (which have also been tested only on older people [4, 10–16]). It appears from our study, that the Fried frailty phenotypes may also be relevant to younger community-dwellers. However, the Fried pre-frail classification requires further examination in younger people to better understand causality and onset of pre-frailty. It may be that requiring one or two components of the Fried frailty phenotype to designate pre-frailty state may be too liberal for people younger than 65 years. If two components, rather than one or two, were required to identify ‘pre-frailty’, this would have reduced the prevalence of pre-frailty to 10.5% in our sample. On the other hand, by identifying the presence of one frailty attribute (any of the Fried frailty criteria), this may assist in identifying people early who are at risk of developing other frailty attributes. We did not test for reliability, and thus we have no evidence of the repeatability over time of the self-report data included in the phenotype (unintentional weight loss, and the amount of physical activity undertaken each week). However, as weight is notoriously under-reported and physical activity is notoriously over-reported [59] it is likely that some of our sample inaccurately estimated usual physical activity patterns, as well as weight change. For instance, the notion of unintentional weight loss may have been lost in our sample in the desire to be seen to be losing weight.

Factor 1 was the best predictor of change in status from not-frail to pre-frail. Risk of early frailty could potentially be reduced by increasing exercise behaviours to improve balance, dynamic stability and muscle strength. The significant predictive capacity of Factors 3, 5 and 7 for pre-frailty to frailty (high psychological distress, living alone, having health worries, and poor sleep quality; stair climbing, appetite, hydration; continence, total food intake) highlights issues which may alter more insidiously than balance, dynamic stability and muscle strength. Given that there was no age difference between pre-frail and frail people (despite the common belief that ageing and frailty is related to body systems decline), it appears that screening people aged 40 years and older not only for physical activity, balance, hearing, foot sensation and muscle strength, but also for mental health, continence, health concerns and poor sleep quality would seem to be important in preventing or delaying frailty onset.

The components of the Fried frailty phenotype, and most variables included in the important predictive factors are potentially modifiable by active interventions. Setting unintentional weight loss aside (which requires medical investigation), our findings suggest that there are many people aged 40 years or older whose frailty status could potentially be addressed by increasing physical activity, building muscle, improving exercise tolerance, nutrition and mental health. The presence of chronic health conditions and concerns about health can be managed actively by supported behaviour change strategies [60]. Reasons for poor foot sensation like diabetes or peripheral neuropathy can be identified following assessment for chronic disease, and solutions for improved foot health and better footwear proposed. Poor hearing can be addressed by audiological or medical intervention and/or hearing aids.

It is reasonable to propose that chronic disease self-management and population health interventions to improve physical activity, such as workplace or community wellbeing programs, could significantly attenuate reverse or slow the onset of pre-frailty in community dwellers aged 40 years or more, and their subsequent risk of progression to frailty [1–3, 6].

### Limitations

#### Interpretation

The authors acknowledge significant potential for respondent bias. Participants were sufficiently literate to read, understand and respond to the recruitment material, and complete the online surveys (98% submitted online). Participants had the time to attend testing and acknowledged strong personal incentives to obtain comprehensive individual health status information, currently unavailable from other sources. It is not known how well these findings reflect people who were less well educated, less health or computer literate, and/or who were not interested in participating in population health screening. Thus, these recruitment strategies, and study findings, require further testing in other community samples. The TMIG study [30] on which our research is partly modelled, recruit participants through the local Tokyo prefecture, using birthdates. The local prefecture office recruit people who have turned 65 years or older since the previous biennial TMIG study. Whilst this rigorous independent recruitment approach has significantly contributed to the size, longevity and impact of the TMIG study, it does not recruit people younger than 65 years. Our multi-pronged recruitment approaches, and strong community partnerships, provided rare access to younger people who would not normally make themselves available, or be targeted, for population health screening initiatives. The health assessments available for analysis in this study while comprehensive were not exhaustive and other factors such as employment status, social connectedness and oral health could be included for future analysis.

#### Generalisability

The study methodology was successful in recruiting a robust sample of volunteers aged 40 to 75 years, from a range of postcodes in one Australian capital city. The sample age, gender and socioeconomic index distribution is thus generalizable to other urban Australians [61, 62]. The similarity in findings of pre-frailty in community dwellers over 50 years in our study with UK [24], English [25], European [26] and Taiwanese [27] studies supports the believability of our findings, particularly as our sample reflects people who are notoriously difficult to comprehensively recruit for community-based population screening [62].

## Conclusion

This paper describes frailty and pre-frailty in community dwellers aged 40 to 75 years. It adds new information to the trajectory of age-related functional decline and frailty in Australia. The findings are concerning, given the lack of gender or age influence on pre-frailty and frailty states. The pre-frailty predictors are largely mutable, and thus potentially amenable to population interventions to improve health behaviours, and halt or reverse poor health outcomes. This study suggests that successful healthy aging interventions should commence in at least the fourth decade of life.

## Data Availability

This data set is still being used for analysis. Please contact the corresponding author regarding access to the full dataset.

## Abbreviations

ABS: Australian Bureau of Statistics
ANOVA: Analysis of variance
AUC: Area under the curve
BMI: Body mass index
CFS: Clinical frailty scale
CI: Confidence interval
F: Female
FiND: Frail non-disabled
FMS: Functional movement screen
M: Male
N: Number
PRISMA-7: Program of research on integration of services for the maintenance of autonomy − 7
ROC: Receiver operator characteristic
SAS: Statistical analysis system
SD: Standard deviation
SPSS: Statistical package for the social sciences
STROBE: Strengthening the reporting of observational studies in epidemiology
UK: United Kingdom
WHO: World Health Organization
Y: Years
